# Gestational Diabetes and Hypothyroidism: Do Other Factors Play a Role? A Retrospective Case–Control Study

**DOI:** 10.1155/nrp/8165809

**Published:** 2026-06-24

**Authors:** Iman Taghizadeh Firoozjaei, Zahra Sabzi, Mohammadreza Rajabloo, Najmeh Javan Sangani, Seyed Ali Kalali Sani, Fatemeh Ranjbar Noei

**Affiliations:** ^1^ Department of Medical-Surgical Nursing, Faculty of Nursing and Midwifery, Golestan University of Medical Sciences, Gorgan, Iran, goums.ac.ir; ^2^ Faculty Member, Nursing Research Center, Golestan University of Medical Sciences, Gorgan, Iran, goums.ac.ir; ^3^ Department of Nursing, Faculty of Nursing and Midwifery, Nursing Research Center, Golestan University of Medical Sciences, Gorgan, Iran, goums.ac.ir; ^4^ Nursing Clinical Research unit, Torbat Jam Faculty of Medical Sciences, Torbat Jam, Iran; ^5^ Student Research Committee, Faculty of Nursing and Midwifery, Golestan University of Medical Sciences, Gorgan, Iran, goums.ac.ir

**Keywords:** gestational diabetes, hypothyroidism, pregnancy

## Abstract

**Background:**

During pregnancy, extensive hormonal and metabolic changes can occur. Gestational diabetes can cause maternal and neonatal complications. Follow‐ups have shown that women who first develop metabolic diseases during pregnancy have an increased risk of developing other metabolic diseases, including hypothyroidism. There are contradictory results. The aim of this study is to provide an overview of the relationship between gestational diabetes and gestational thyroid dysfunctions.

**Methods:**

This study is a retrospective case–control study conducted in 2025 on 353 pregnant women in Iran; 176 were included in the case group (women with gestational diabetes) and 177 in the control group (women without gestational diabetes). The diagnosis of hypothyroidism was assessed in both groups. Descriptive statistics, chi‐square test, *t*‐test, and unconditional logistic regression were used to analyze the data.

**Findings:**

Gestational diabetes was associated with a significant increase in the risk of hypothyroidism (approximately 2.8‐fold) (*p* = 0.03). A history of other diseases was also associated with a significantly increased risk of developing hypothyroidism (*p* = 0.001). In contrast, the risk of hypothyroidism was not significantly associated with age at first pregnancy (*p* = 0.10), number of pregnancies (*p* = 0.11), or breastfeeding (*p* = 0.35).

**Conclusion:**

Gestational diabetes and a history of other diseases significantly increased hypothyroidism risk, while age at first pregnancy, number of pregnancies, and breastfeeding showed no significant associations. Based on these findings, regular thyroid screening is suggested for pregnant women with gestational diabetes or other medical conditions, regardless of reproductive factors.

## 1. Introduction

During pregnancy, extensive hormonal changes occur (glucose homeostasis and thyroid function adaptation) that require the occurrence of several compensatory mechanisms to establish the new balances caused by pregnancy [1]. Accordingly, pregnancy can be considered a challenging event, where even a small reduction in prepregnancy functional reserve (i.e., insulin resistance or decreased thyroid reserve) may lead to impaired adaptive responses and subsequent pathological states. GDM^1^ and GTD^2^ are the most common endocrine disorders during pregnancy [1, 2].

GDM is an abnormal glucose metabolism that first develops during pregnancy [3]. The incidence of GDM is increasing worldwide, especially in low‐income and developing countries [4–6]. It is the most common complication during pregnancy with a prevalence of 9.4%–10.6% and is associated with many adverse fetal and maternal outcomes [7]. It can cause maternal and neonatal complications such as preeclampsia, preterm delivery, and an increased risk of long‐term endocrine disorders [6]. The Iranian Ministry of Health has emphasized in its latest guidelines the need to screen pregnant women with a 75‐g OGTT^3^ between 24 and 28 weeks of pregnancy [8]. Long‐term follow‐ups have shown that women who first develop metabolic diseases during pregnancy have an increased risk of developing other metabolic diseases, including Type 2 diabetes or hypothyroidism [7].

Thyroid hormones are important for the current and long‐term health of all humans, including mothers and children [8]. Their roles include organ and neuronal development, reproduction, and regulation of metabolism and energy [9]. Hypothyroidism is a biochemical diagnosis in which the thyroid gland is unable to produce sufficient amounts of thyroid hormone to meet the body’s metabolic needs [10]. Subclinical hypothyroidism may progress to overt hypothyroidism in approximately 2%–5% of cases per year [11]. Symptoms of hypothyroidism may include adverse effects and outcomes such as an increased risk of coronary artery disease and cardiovascular mortality [11], fatigue, cold intolerance, weight gain, constipation, and decreased mood, quality of life, cognitive function, and memory [12], which can be significantly reduced with effective treatment [11]. In a study conducted by Prasad et al., the incidence of gestational diabetes in pregnant women with hypothyroidism was 8% and, in the control group, it was 1% [13]. In contrast, the study by Sharifi et al. reported a lower odds ratio (OR) of individuals with GDM to have clinical hypothyroidism than the control group [14].

These two diseases (GDM and GTD) may lead to the development or progression of adverse maternal and fetal outcomes (such as preeclampsia, preterm delivery, and an increased risk of long‐term endocrine disorders) and long‐term complications in the postpartum period. Given the potential impact of these conditions on pregnancy outcomes and considering the conflicting evidence in the literature, this study aims to investigate these two outcomes during pregnancy and their relationship with other factors such as disease history, breastfeeding status, and age at first pregnancy, which have been less frequently mentioned in other studies.

## 2. Methodological Approach

This study is a retrospective study conducted on 353 pregnant women living in Golestan Province (a province in northern Iran). Data were collected from two university‐affiliated prenatal clinics Gorgan, Iran. These centers were selected based on convenience and the availability of complete electronic medical records for the study period in 2025.

### 2.1. Participants and Research Population

To obtain information, the records of individuals referring to centers affiliated with Golestan University of Medical Sciences who met the inclusion criteria for our study were used. The records were reviewed from 2014 to 2025. According to the results of similar studies [7], with a confidence level of 95%, power of 80%, two‐sided test, two‐proportion comparison formula, and using STATA Version 11 software, the sample size was estimated to be 176 people in each group. Our study population included all women who met the inclusion criteria, were pregnant, had gestational diabetes (the case group), and pregnant women who did not have gestational diabetes (the control group), and the incidence of hypothyroidism was assessed in these two groups (both preexisting and gestational hypothyroidism were included).

#### 2.1.1. Inclusion Criteria

1. Women between 15 and 45 years.
2. Those who had the information required for the study in their files.
3. The case group consisted of singleton pregnant women with gestational diabetes, and the control group consisted of singleton pregnant women without gestational diabetes. Then, the incidence of hypothyroidism was compared between the two groups. GDM was diagnosed according to the International Association of Diabetes and Pregnancy Study Groups (IADPSG) criteria: fasting plasma glucose ≥ 92 mg/dL, 1‐h glucose ≥ 180 mg/dL, or 2‐h glucose ≥ 153 mg/dL following a 75‐g OGTT [8].

#### 2.1.2. Exclusion Criteria

✓ Absence of required information in the records.
✓ Presence of other serious diseases (including types of cancer and cardiovascular, pulmonary, and renal diseases) and people taking medications that increase blood sugar (such as corticosteroids, cyclosporine, etc.).
✓ People with a history of preexisting diabetes.

### 2.2. Ethical Consideration

This *research project* was initially approved by the Ethics Committee of Golestan University of Medical Sciences under the number of project: IR.GOUMS.REC.1403.243.

### 2.3. Sampling and Randomization

To conduct sampling, a list of health and medical centers was prepared. Then, the researcher visited the health and medical centers and, after explaining the goals and gaining their trust, began sampling. An initial checklist was completed to apply the inclusion and exclusion criteria and select eligible samples for each sample. The samples of the case group were selected based on the inclusion criteria until the predicted number was reached. After that, for each sample selected in the case group, one sample was randomly selected from other individuals referring to the centers for the control group. It should be noted that when selecting individuals for the case and control groups, they were assessed based on age group, body mass index, place of residence, and history of illness (autoimmune disease history) through group comparability, and no significant differences were found.

### 2.4. Data Analysis

The collected data were entered into SPSS Version 21. First, the normality of the data was examined using the Kolmogorov–Smirnov test. Descriptive statistics were used to estimate the mean, frequency, and percentage. To determine the relationship between demographic characteristics and hypothyroidism and the relationship between hypothyroidism and gestational diabetes, the *t*‐test, chi‐square test, unconditional logistic regression, OR, and confidence interval (CI) were used. The results were reported following the Strengthening the Reporting of Observational studies in Epidemiology (STROBE) checklist [15]. *p* values < 0.05 were considered significant. For multivariate analysis, variables with *p* < 0.20 in univariate analysis or known clinical relevance were entered into the logistic regression model using a forward stepwise method (entry criterion *p* < 0.05, removal criterion *p* > 0.10). Model goodness‐of‐fit was assessed using the Hosmer–Lemeshow test. Multicollinearity was assessed using the variance inflation factor (VIF). All VIF values were below 5, indicating no significant collinearity among the predictors. All results are reported as ORs with 95% CIs.

## 3. Results

This study was conducted on 176 pregnant women in the case group and 177 pregnant women in the control group. Table 1 indicates that variables such as occupation (*p* = 0.002), age at first pregnancy (< 0.001), number of pregnancies (*p* = 0.04), and deliveries (*p* = 0.01) have significant differences between the two groups, which is associated with the incidence of hypothyroidism. Other variables (such as age, BMI, education, place of residence, history of illness [history of autoimmune disease was recorded as part of the history of illness] [*p* = 0.21], substance use [including both smoking, cigarette or hookah, and illicit drug use], etc.) did not show a statistically significant difference (*p* > 0.05).

**TABLE 1:** Demographic and characteristics of case and control groups.

Group variable	Case (*n* = 176)	Control (*n* = 177)	*p* value	(95% CI: lower–upper)
Age (y)	45.80 ± 8.17	44.71 ± 9.33	0.24^∗^	[38.28, 40.87]
BMI (kg/m^2^)	28.21 ± 4.99	27.73 ± 7.86	0.73^∗^	[19.59, 35.59]
Education				
Below diploma	75	131		—
Diploma	53	27	0.83^∗∗^	
Academic	49	18		
Place of residence				
Urban	110 (62.5%)	107 (60.5%)		—
Rural	66 (37.5%)	70 (39.5%)	0.77^∗∗^	
History of illness (rheumatoid arthritis, systemic lupus erythematosus, Hashimoto’s thyroiditis, autoimmune disease, cancer, cardiovascular, and pulmonary and renal disease)				
Yes	77 (43.8%)	90 (50.8%)		—
No	99 (56.2%)	87 (49.2%)	0.21^∗∗^	
Marital status				
Married	173 (98.3%)	176 (99.4%)		—
Single	3 (1.7%)	1 (0.6%)	0.61^∗∗^	
Substance use history/smoking				
Yes	4 (2.3%)	8 (4.5%)		—
No	171 (97.7%)	170 (95.5%)	0.38	
Occupation				
Yes	19 (10.8%)	42 (23.7%)		—
No	157 (89.2%)	135 (76.3%)	0.002^∗∗^	
Age at first pregnancy	21.01 ± 6.12	23.62 ± 4.65	< 0.001^∗^	[22.42, 23.91]
Breastfeeding history				
Yes	159 (90.3%)	149 (85.1%)		—
No	17 (9.7%)	26 (14.9%)	0.18^∗∗^	
Number of pregnancies	2.77 ± 1.53	2.43 ± 1.54	0.04^∗^	[2.52, 2.85]
Number of deliveries	2.35 ± 1.35	1.99 ± 1.30	0.01^∗^	[1.72, 2.01]

*Note:* Statistical analyses were performed using the independent *t*‐test. *p* < 0.05 was considered statistically significant.

^∗^for continuous variables and the chi‐square test.

^∗∗^for categorical variables.

Table 2 shows that the number of people in the case group with hypothyroidism was 35, while there were 6 in the control group. Also, the number of people without hypothyroidism was 151 in the case group and 183 in the control group. The results showed that the incidence of hypothyroidism in the case group was significantly higher than the control group (*p* = 0.02). This finding could indicate a possible association between gestational diabetes and the incidence of hypothyroidism.

**TABLE 2:** Comparison of the incidence of hypothyroidism among the case and control groups.

Group variable	Case (*n* = 176)	Control (*n* = 177)	*p* value	OR (95% CI: lower–upper)
Hypothyroidism				
Yes	35 (18.8%)	6 (3.2%)	0.02^∗∗^	[1.12, 7.33]
No	151 (81.2%)	183 (96.8%)		

*Note:* Data are presented as mean ± SD or *n* (%).

^∗∗^Chi‐square test. *p* < 0.05 was considered statistically significant.

The Hosmer–Lemeshow test indicated good model fit (*χ* ^2^ = [7.299], df = 8, *p* = [0.565]), confirming the adequacy of the logistic regression model. Multicollinearity was assessed using the VIF. All VIF values ranged from 1.018 to 1.203, which are below the threshold of 5, indicating no significant multicollinearity among the predictors. Table 3 reveals that women with a history of gestational diabetes are approximately 2.8 times more likely to develop hypothyroidism; this is also statistically significant (*p* = 0.03). For each year of age at first pregnancy, the risk of hypothyroidism increases by about 4.6%; this association is not significant (*p* = 0.10). The risk of hypothyroidism decreases with increasing number of pregnancies, but this result is not significant (*p* = 0.11). This finding may be affected by confounding factors (such as maternal age). Breastfeeding is associated with a 63% increased risk of hypothyroidism, but the CI is very wide (*p* = 0.35), suggesting that this association is not statistically significant. Breastfeeding itself is not a significant risk factor. People with a history of other diseases are 3.8 times more likely to have hypothyroidism, which is also statistically significant (*p* = 0.001). Although some CIs (e.g., for the breastfeeding variable: OR =  95% CI: [0.576–4.614]) were wide, this likely reflects limited statistical power for secondary analyses rather than the absence of an effect. The primary finding for GDM and hypothyroidism demonstrated acceptable precision OR = [95% CI: [1.093–7.161].

**TABLE 3:** Unconditional logistic regression of hypothyroidism with other variables.

Variable	Odds ratio (OR)	*p* value	VIF	(95% CI: lower–upper)
Gestational diabetes	2.798	0.032	1.127	[1.093, 7.161]
Age at first pregnancy	1.046	0.102	1.130	[0.991, 1.105]
Number of pregnancies	0.760	0.119	1.203	[0.538, 1.073]
Breast feeding history	1.630	0.357	1.181	[0.576, 4.614]
History of illness	3.839	0.001	1.048	[1.687, 8.740]
Occupation	0.750	0.598	1.018	[0.025, 2.192]

*Note:* Data are presented as odds ratio (OR) with 95% confidence intervals (CI) and corresponding *p* values. OR > 1 indicates increased risk, and OR < 1 indicates decreased risk. *p* values were calculated using logistic regression. A *p* value < 0.05 was considered statistically significant.

## 4. Discussion

### 4.1. This Study Examined the Relationship Between GDM and Hypothyroidism

Women with a history of gestational diabetes are approximately 2.8 times more likely to develop hypothyroidism. Several mechanisms may explain the observed association between GDM and hypothyroidism. First, insulin resistance in GDM alters placental deiodinase activity, reducing DIO2 (which converts T4 to active T3) and increasing DIO3 (which inactivates thyroid hormones), which disrupts thyroid hormone metabolism [16]. Second, chronic low‐grade inflammation evidenced by elevated TNF‐α, IL‐6, and hs‐CRP in GDM can suppress the hypothalamic–pituitary–thyroid axis [17]. Third, pregnancy increases thyroid hormone demand, and the metabolic stress of GDM may unmask preexisting subclinical hypothyroidism [18]. Additionally, large‐scale evidence suggests that lower maternal FT4 levels independently increase GDM risk, indicating a bidirectional relationship [19]. Further mechanistic studies are needed to establish causality, which was consistent with the findings of the study by Sert, Safian, and Gong [10, 20, 21]. Also, the findings of this study were contrary to the findings of Sharifi et al. because the results of their study showed that thyroid dysfunction is not associated with gestational diabetes [14]. Therefore, GDM was associated with an increased prevalence of hypothyroidism; however, causality cannot be inferred. Prospective studies are needed to determine whether GDM precedes and predicts hypothyroidism. On the other hand, physicians should be aware of the possibility of the occurrence or progression of hypothyroidism in women with gestational diabetes. This study showed that demographic characteristics such as age and body mass index did not differ significantly between the case and control groups, which was in line with the study by Moosazadeh et al. [7], indicating that the selection of samples in the two groups was done correctly. In this study, it was found that the variables of substance use and disease history were significantly different between the two groups and were likely associated with the occurrence of hypothyroidism. These results are in line with the study by Zahedi et al.[22], who reported the association between substance use and thyroid disorders. The possible mechanism of this association could be due to the effect of drugs on the hypothalamic–pituitary–thyroid axis; overall, these results emphasize the importance of screening for hypothyroidism in individuals with a history of substance use or related diseases. For each year of increasing age at first pregnancy, the risk of hypothyroidism increased by about 4.6%, but this association was not statistically significant; this is consistent with our results and with those in another study by Lean et al.[23], which reported a weak association between age at first pregnancy ≥ 30 years and thyroid disorders, which may indicate that age alone is not an independent risk factor and needs to interact with other factors (such as a history of autoimmune diseases or BMI). Hochler et al.’s [24] study also showed that older maternal age (especially first pregnancy at age ≥ 35 years) is associated with an increased risk of hypothyroidism but emphasizes that other factors such as BMI and a history of autoimmune diseases also play a role, probably due to differences in controlling for confounders. To clarify this association, it is suggested that future studies specifically examine women over 35 years of age by carefully measuring inflammatory and autoimmune markers. In the present data analysis, a trend toward a decreased risk of hypothyroidism with an increasing number of pregnancies was observed, although this association was not statistically significant. This result may have been influenced by confounding factors such as maternal age or autoimmune status. Consistent with our results, Zabuliene et al.[25] reported that women with 3 or more pregnancies had a 15% reduced risk of hypothyroidism compared with nulliparous women. This association was also not statistically significant, and the researchers suggested that it may be due to the modulation of the immune system in multiple pregnancies. Another study by Floriani et al.[26] showed that each additional pregnancy was associated with an 8% increased risk of hypothyroidism, which emphasized the cumulative physiological stress on the thyroid gland in multiple pregnancies, and the difference in results may be due to differences in the study population. Therefore, although the evidence on the relationship between the number of pregnancies and the risk of hypothyroidism is contradictory, these contradictions reflect more the complexity of this relationship and the influence of various underlying factors than the methodological weaknesses of the studies. To clarify this relationship, more rigorous research with a longitudinal design and more careful control of confounding variables is needed. In the present study, although breastfeeding was associated with a 63% increased risk of hypothyroidism, this association was not statistically significant, and the CI was very wide, indicating that this finding is likely to be due to chance and that breastfeeding cannot be considered an independent risk factor for hypothyroidism. This spurious association may be due to confounding factors such as postpartum hormonal changes or metabolic stress, which are associated with both breastfeeding and thyroid function, or it may be due to maternal nutritional status (particularly iodine and selenium intake), which can affect both breastfeeding and thyroid function. Consistent with our results, the study by Lopes et al.[16] also showed that breastfeeding was not significantly associated with thyroid dysfunction at 12‐month postpartum follow‐up. From a nursing perspective, the findings suggest that pregnant women with GDM should be monitored more closely for thyroid dysfunction. Nurses can play a key role by (1) assessing for signs and symptoms of hypothyroidism in GDM patients, (2) coordinating timely thyroid function testing, and (3) educating women about the potential coexistence of these two conditions [27, 28]. Beyond statistical significance, the clinical importance of our findings should be considered. The observed association between GDM and hypothyroidism yielded an OR of approximately 2.798, indicating that women with GDM are nearly three times more likely to have hypothyroidism compared to those without GDM. This effect size is clinically meaningful. From a nursing and clinical practice perspective, this magnitude of association justifies closer monitoring of thyroid function in pregnant women diagnosed with GDM. For example, routine thyroid function testing (TSH and free T4) could be considered at the time of GDM diagnosis and again in the early postpartum period. In contrast, variables with wide CIs (such as breastfeeding) produced imprecise estimates. While these results did not reach statistical significance, the wide intervals suggest that our study had limited power to detect or rule out meaningful associations for these secondary variables. Therefore, null findings for secondary variables should be interpreted as inconclusive rather than evidence of no effect.

### 4.2. Limitation

The samples were selected from centers affiliated with Golestan University of Medical Sciences, which may not be fully representative of the general population (for example, women with less access to health centers may not have been included in the study).

We did not have data on urinary iodine or dietary intake.

## 5. Conclusion

The present study, which was conducted on two groups of pregnant women, showed that GDM and a history of other diseases were significantly associated with an increased risk of hypothyroidism. The OR of approximately 2.8 indicates that women with GDM are nearly three times more likely to develop hypothyroidism compared to those without GDM, which is a clinically meaningful effect size. In contrast, the association of factors such as age at first pregnancy, number of pregnancies, and breastfeeding with the risk of hypothyroidism was not statistically significant, and the wide CIs for some of these variables suggest limited statistical power for secondary analyses. These findings emphasize the importance of paying attention to thyroid status in pregnant women with a history of gestational diabetes or other comorbidities. From a nursing perspective, routine thyroid function testing (TSH and free T4) is recommended at the time of GDM diagnosis and during prenatal follow‐up. Further prospective studies with larger sample sizes are needed to investigate the role of other related factors and to confirm the exploratory findings of this study.

## Funding

This study was funded by the Golestan University of Medical Sciences, Gorgan, Iran.

## Conflicts of Interest

The authors declare no conflicts of interest.

## Endnotes

^1^Gestational diabetes mellitus.

^2^Gestational thyroid dysfunctions.

^3^Oral glucose tolerance test.

## Data Availability

The data that support the findings of this study are available from the corresponding author upon reasonable request.
